# Supplementary material for: Nutritional status, dementia, and mobility among nursing home’s residents: First exhaustive cross-sectional study in Limousin territory (France)
Source: PLoS One. 2021 Apr 30;16(4):e0250595. doi: 10.1371/journal.pone.0250595 (PMC8087088; doi:10.1371/journal.pone.0250595)
Supplement: Attachment — Submitted filename: PONE-D-20-40393_reviewer.pdf [file pone.0250595.s001.pdf]

PLOS ONE

Nutritional status, dementia and mobilityamongnursing home's residents:First exhaustive cross-sectional study in Limousin Territory (France)

--Manuscript Draft--

**Manuscript Number:**	PONE-D-20-40393
**Article Type:**	Research Article
**Full Title:**	Nutritional status, dementia and mobilityamongnursing home's residents:First exhaustive cross-sectional study in Limousin Territory (France)
**Short Title:**	Nutritional status, dementia and mobility among nursing home's residents
**Corresponding Author:**	Gustave Mabiama, MSc<br>Limoges University: Universite de Limoges<br>Limoges, Nouvelle-Aquitaine FRANCE
**Keywords:**	nursing home; undernutrition; dementia; mobility; Elderly
**Abstract:**	BACKGROUND: Aging is accompanied by a drop in the level of health and autonomy; within Western countries more and more people being cared for in nursing homes (NH). The nutritional data in NH in France remain poor, not exhaustive and not representative. The objective of the study was to assess the nutritional status, dementia and mobility patterns among residents of NH in the Limousin territory of France.<br>METHODS: The study was cross-sectional, descriptive and exhaustive, conducted with the residents of 13 voluntary NH. Undernutrition was identified using French High Authority for Health criteria, and obesity if Body Mass Index >30, in the absence undernutrition criterion. The Mini Mental State was used for dementia assessment at the threshold of 24. The Mini Nutritional Assessment TM was used for mobility assessment. Significant statistics at the 5% threshold.<br>RESULTS: 866 residents (70.6% women) included with an average age of 85.3 ± 9.3 years. Undernutrition was 27.5%, obesity 22.9%, dementia 45.7% and very low mobility 68.9%. Women were older than men, more often undernourished, more often demented and more often had very low mobility (p<0.01). Undernutrition (p<0.0001) and low mobility (p<0.0001) were significantly higher among dementia versus those without dementia. Very low mobility was higher among undernourished (p<0.05).<br>CONCLUSIONS: Undernutrition and obesity are important problems in NH in France. Being a woman, having dementia and having a very low mobility may induce undernutrition.
**Order of Authors:**	Philippe Fayemendy
	Thibault Vernier
	Gustave Mabiama, MSc
	Aude Massoulard-Gainant
	Carole Villemonteix
	Jean-Claude Desport
	Pierre Jesus
**Additional Information:**	
**Question**	**Response**
**Financial Disclosure**<br><br>Enter a financial disclosure statement that describes the sources of funding for the work included in this submission. Review the submission guidelines for detailed requirements. View published research	The author(s) received no specific funding for this work.

Powered by Editorial Manager® and ProduXion Manager® from Aries Systems Corporation

articles from *PLOS ONE* for specific examples.<br><br>This statement is required for submission and **will appear in the published article** if the submission is accepted. Please make sure it is accurate.<br><br>**Unfunded studies**<br>Enter: *The author(s) received no specific funding for this work.*<br><br>**Funded studies**<br>Enter a statement with the following details:<br>• Initials of the authors who received each award<br>• Grant numbers awarded to each author<br>• The full name of each funder<br>• URL of each funder website<br>• Did the sponsors or funders play any role in the study design, data collection and analysis, decision to publish, or preparation of the manuscript?<br>• **NO** - Include this sentence at the end of your statement: *The funders had no role in study design, data collection and analysis, decision to publish, or preparation of the manuscript.*<br>• **YES** - Specify the role(s) played.<br><br>* typeset	
**Competing Interests**<br><br>Use the instructions below to enter a competing interest statement for this submission. On behalf of all authors, disclose any competing interests that could be perceived to bias this work—acknowledging all financial support and any other relevant financial or non-financial competing interests.<br><br>This statement **will appear in the published article** if the submission is accepted. Please make sure it is accurate. View published research articles from *PLOS ONE* for specific examples.	NO authors have competing interests

Powered by Editorial Manager® and ProduXion Manager® from Aries Systems Corporation

**NO authors have competing interests**<br><br>Enter: *The authors have declared that no competing interests exist.*<br><br>**Authors with competing interests**<br><br>Enter competing interest details beginning with this statement:<br><br>*I have read the journal's policy and the authors of this manuscript have the following competing interests: [insert competing interests here]*<br><br>* typeset	
**Ethics Statement**<br><br>Enter an ethics statement for this submission. This statement is required if the study involved:<br><br>• Human participants<br>• Human specimens or tissue<br>• Vertebrate animals or cephalopods<br>• Vertebrate embryos or tissues<br>• Field research<br><br>Write "N/A" if the submission does not require an ethics statement.<br><br>General guidance is provided below. Consult the submission guidelines for detailed instructions. **Make sure that all information entered here is included in the Methods section of the manuscript.**	The study was validated by the Ethics Committee and the Board of Directors of the LINUT Network, a structure of the Agence Régionale de Santé Nouvelle Aquitaine, in accordance with the agreement signed with the nursing homes for the use of data collected for scientific purposes, after acceptance by the residents or their legal representatives.

Powered by Editorial Manager® and ProduXion Manager® from Aries Systems Corporation

**Format for specific study types**<br><br>**Human Subject Research (involving human participants and/or tissue)**<br>• Give the name of the institutional review board or ethics committee that approved the study<br>• Include the approval number and/or a statement indicating approval of this research<br>• Indicate the form of consent obtained (written/oral) or the reason that consent was not obtained (e.g. the data were analyzed anonymously)<br><br>**Animal Research (involving vertebrate animals, embryos or tissues)**<br>• Provide the name of the Institutional Animal Care and Use Committee (IACUC) or other relevant ethics board that reviewed the study protocol, and indicate whether they approved this research or granted a formal waiver of ethical approval<br>• Include an approval number if one was obtained<br>• If the study involved *non-human primates*, add *additional details* about animal welfare and steps taken to ameliorate suffering<br>• If anesthesia, euthanasia, or any kind of animal sacrifice is part of the study, include briefly which substances and/or methods were applied<br><br>**Field Research**<br><br>Include the following details if this study involves the collection of plant, animal, or other materials from a natural setting:<br>• Field permit number<br>• Name of the institution or relevant body that granted permission	
**Data Availability**<br><br>Authors are required to make all data underlying the findings described fully available, without restriction, and from the time of publication. PLOS allows rare exceptions to address legal and ethical concerns. See the PLOS Data Policy and FAQ for detailed information.	No - some restrictions will apply

A Data Availability Statement describing where the data can be found is required at submission. Your answers to this question constitute the Data Availability Statement and **will be published in the article**, if accepted.<br><br>**Important:** Stating 'data available on request from the author' is not sufficient. If your data are only available upon request, select 'No' for the first question and explain your exceptional situation in the text box.<br><br>Do the authors confirm that all data underlying the findings described in their manuscript are fully available without restriction?	
**Describe where the data may be found in full sentences. If you are copying our sample text, replace any instances of XXX with the appropriate details.**<br><br>• If the data are **held or will be held in a public repository**, include URLs, accession numbers or DOIs. If this information will only be available after acceptance, indicate this by ticking the box below. For example: *All XXX files are available from the XXX database (accession number(s) XXX, XXX.*).<br>• If the data are all contained **within the manuscript and/or Supporting Information files**, enter the following: *All relevant data are within the manuscript and its Supporting Information files.*<br>• If neither of these applies but you are able to provide **details of access elsewhere**, with or without limitations, please do so. For example:<br><br>*Data cannot be shared publicly because of [XXX]. Data are available from the XXX Institutional Data Access / Ethics Committee (contact via XXX) for researchers who meet the criteria for access to confidential data.*<br><br>*The data underlying the results presented in the study are available from (include the name of the third party*	Availability of supporting data: Under the agreements signed between Elderly Nursing homes and the Limousin Nutrition network (LINUT), health network of the Nouvelle Aquitaine Regional Health Agency, data are collected, saved and protected within the LINUT network.

and contact information or URL).<br>• This text is appropriate if the data are owned by a third party and authors do not have permission to share the data.<br><br>* typeset	
Additional data availability information:	

Powered by Editorial Manager® and ProduXion Manager® from Aries Systems Corporation

Manuscript Click here to access/download;Manuscript;Nutritional status in nursing homes.docx

Nutritional status, dementia, and mobility among nursing home's residents: First exhaustive cross-sectional study in Limousin Territory (France)

Philippe Fayemendy (MD)[1,2,3], Thibault Vernier (MD)[1], Gustave Mabiama (MSc)[1,2,4,*], Aude Massoulard-Gainant (MD)[3,5], Carole Villemonteix (BBA)[3], Jean-Claude Desport (PhD)[1,2,3], Pierre Jésus (PhD)[1,2,3]

[1]Nutrition Unit and Specialized Centre for Obesity of the Limousin territory, University Hospital, avenue Martin Luther King, 87042, Limoges cedex, France

[2]Inserm U1094, Limoges university, IRD, Tropical Neuroepidemiology, Epidemiology and Neurology Tropical Institute, GEIST, rue du Dr Marcland, 87000 Limoges, France

[3]Health Regional Agency network Limousin Nutrition (LINUT), 16 rue du Cluzeau ; 87270 Isle, France

87042, Limoges cedex, France

[4]Microbiology, Immunology-Hematology and Morphologic Sciences Laboratory (LMIHSM), Doctoral Training Unit in Health Sciences (UFD-SCS), Doctoral School, Douala University, BP 2701, Douala, Cameroun

[5]Home hospitalization and Geriatric Service, University Hospital, avenue Martin Luther King,

*Corresponding author: gustave.mabiama@unilim.fr

Abstract

BACKGROUND: Aging is accompanied by a drop in the level of health and autonomy; with in Western countries more and more people being cared for in nursing homes (NH). The nutritional data in NH in France remain poor, not exhaustive and not representative. The objective of the study was to assess the nutritional status, dementia and mobility patterns among residents of NH in the Limousin territory of France.

METHODS: The study was cross-sectional, descriptive and exhaustive, conducted with the residents of 13 voluntary NH. Undernutrition was identified using French High Authority for Health criteria, and obesity if Body Mass Index >30, in the absence undernutrition criterion. The Mini Mental State was used for dementia assessment at the threshold of 24. The Mini Nutritional Assessment™ was used for mobility assessment. Significant statistics at the 5% threshold.

RESULTS: 866 residents (70.6% women) included with an average age of 85.3 ± 9.3 years. Undernutrition was 27.5%, obesity 22.9%, dementia 45.7% and very low mobility 68.9%. Women were older than men, more often undernourished, more often demented and more often had very low mobility ($p<0.01$). Undernutrition ($p<0.0001$) and low mobility ($p<0.0001$) were significantly higher among those with dementia versus those without dementia. Very low mobility was higher among undernourished ($p<0.05$).

CONCLUSIONS: Undernutrition and obesity are important problems in NH in France. Being a woman, having dementia and having a very low mobility may induce undernutrition.

Key words: nursing home, undernutrition, dementia, mobility, elderly

Highlights:

- In nursing home undernutrition was found in 27.5% of cases.
- In nursing home obesity was found in 22.9% of cases.
- Undernutrition and mobility were significantly higher among demented persons.
- Very low mobility was higher among undernourished

Introduction

In France, the aging of the population and the increase in dependence of the oldest people led between 2008 and 2016 to a 2.7% increase per year in the number of places in NH, which in 2017 reached 605,000 (1). Nutritional problems are notable in these establishments, both in terms of undernutrition and obesity.

Undernutrition in the elderly has multiple consequences in terms of morbidity (increased risk of infection, fall, pressure ulcer, bone embrittlement, deterioration of the psychological state, reduction of autonomy and quality of life, etc.), and constitutes a risk factor for mortality in NH (2-4). The prevalence of undernutrition is evaluated in France up to 10% for the elderly at home and up to 40% for residents of nursing homes (4). At the international level, the range in nursing homes is wider (1.5-66.5%), depending on the populations studied and the tools used, with an average estimated at 20%.

Although obesity in the elderly has sometimes been considered favorable from the point of view of mortality or morbidity (obesity paradox) (5), for many pathologies moderate or severe obesity seems deleterious, with in particular an increase in the risk of falls, arthritis, diabetes, bedsores, a reduction in autonomy (6-9). Its prevalence in French NH is not known, but French data in the general population show that the prevalence of obesity increases with age, at least up to 75 years (10). Data in the United States indicates an increase in the prevalence of obesity in NH between 1992 (15%) and 2015 (27.9%) (9).

Both undernutrition and obesity are associated in EHPAD with an increase in the demand for care, therefore at increased personnel and material costs, and this increase is particularly marked among obese residents (8,11).

Furthermore, the link between dementia and undernutrition or the risk of undernutrition is well known (12-15), as well as the link between low mobility and obesity (12) or undernutrition and reduced physical capacity (16).

The nutritional data in nursing homes are very disparate with sometimes a lack of completeness and / or representativeness, whether they come from one or more nursing homes (Table 1) (2,3,9,15,16), and the prevalence figures specifically for France are more evaluations or expert consensus than confirmed figures (Table 1) (17,18).

The Limousin Nutrition network (LINUT) is a structure of the Regional Health Agency (ARS) of the New Aquitaine Region in France. Since 2004, this network has been providing nutritional care in nursing homes, and has a preventive role in these establishments (19).

The objective of the study was to analyze the data of exhaustive nutritional evaluation surveys carried out between 2008 and 2014 in NH in three departments of the New Aquitaine region, in connection with the presence of dementia and with the mobility of the residents.

Methods

The exhaustive descriptive cross-sectional study covered 13 NH in the Limousin territory (including three departments and around 800,000 inhabitants), between 2008 and 2014. The NH were all volunteers, they were contracted for clinical research with the LINUT network, and residents or their having rights had given their informed consent for the assessments.

All residents of each NH were included, except those whose state of health, according to the doctor, did not allow the measurements or those who refused the measurements after being informed. The assessments were carried out by a doctor and a dietician from the network specializing in geriatrics, and lasted two to three days by EHPAD, depending on the number of residents to be assessed.

Residents' weight was measured in underwear using an electronic scale (SECA 813, Hamburg, Germany, to the nearest 0.1 kg) or an electronic weighing pan (SECA 634, Hamburg, Germany, to the nearest 0.1 kg or A&Z Gastineau, Roissy, France, to the nearest 0.1 kg). The size was measured using a wall measurement to the nearest 0.1 cm. For patients who cannot be verticalized, measuring the heel-to-knee distance using an appropriate measuring rod (Securimed 2068, Cappelle-la-Grande, France, to within 0.5 cm) made it possible to obtain the size using the Chumlea formulas (20). The nutritional status of residents was determined according to the French criteria of the Haute Autorité de Santé (HAS) of 2007 for people over the age of 70 (21), and the 2003 French criteria for people under 70 (22). Undernutrition was present if i) the BMI was <21 for residents over 70 and <18.5 for other residents, or if ii) the weight loss was >=5% in one month or >=10 % in six months, or else iii) the Mini Nutritional Assessment (MNA™) was <17 in residents over 70 years of age. Overweight was defined by the existence, in the absence of a malnutrition criterion, of a BMI between 25 and 29.9 for people under 70 or between 27 and 29.9 for people over 70 years old. Obesity was defined by the existence, in the absence of an undernutrition criterion, of a BMI >=30 whatever the age. For cost reasons and possible differences in normality limits depending on the measurement laboratory, albuminemia was not achieved.

The Mini Mental State (MMS) was used to determine whether or not dementia was present at the threshold of 24 (23). Mobility was assessed using an MNA™ criterion, according to two classes: "very low mobility", corresponding to a bedridden state or the resident's ability to move only from bed to chair, "poor or good mobility" corresponding mobility possible inside and outside the NH. This MNA™ criterion was applied regardless of the age of the residents. The study was validated by the Ethics Committee and the Board of Directors of the LINUT Network, a structure of the Agence Régionale de Santé Nouvelle Aquitaine, in accordance with the agreement signed with the

nursing homes for the use of data collected for scientific purposes, after acceptance by the residents or their legal representatives.

The databases were set up prospectively. They were processed anonymously using Statview™ software (SAS, Cary, USA). The quantitative values are presented as means ± standard deviation and the qualitative values as percentages. The analysis used Fischer's exact test, Student's t test and ANOVA. There was no multivariate analysis, given the limited number of criteria available in NH. The significance threshold was set at p<0.05.

Results

The assessments covered 866 of the 1,043 residents of NHs surveyed (83%), or 66.6±14.7 residents/NH, and 17.0% of the residents could not be assessed either because of their refusal or because of their fragility. The M/F ratio was 0.42, or 70.6% women. The average age of residents was 85.3±9.3 years, the average BMI 26.4±5.3. Dementia was identified in 45.7% of the cases, and 31.1% of the residents were bedridden or could only go from bed to chair. Undernutrition affected 27.5% of residents, obesity 22.9% (Figure 1).

Women were older than men (87.1±8.1 versus 80.7±10.4 years, p<0.0001), more often undernourished than men (30.7% versus 20.0%, p=0.01), with a tendency to be overweight less often (15.0 versus 20.4%, p=0.058) (Table 2). They were more often demented (51.9% versus 30.8%, p<0.0001) and more often had very low mobility (66.3% versus 75.4%, p=0.02).

The nutritional status was different (p<0.0001) depending on the presence or not of dementia (Table 2). Compared to non-demented residents, the demented were more often undernourished (p<0.0001), less often overweight (p=0.01) or obese (p=0.004) and more often very limited in terms of mobility (38.9% of cases versus 23.9%, p<0.0001).

The nutritional status was different ($p<0.0001$) depending on mobility (Table 2). Residents with very low mobility were more often undernourished ($p<0.0001$) less often with normal nutritional status ($p=0.01$) or overweight ($p<0.0001$) than those with poor or good mobility. There was no significant difference for obesity.

There was no significant difference for nutritional status according to NH, either in overall analysis of the four classes of nutritional status ($p=0.62$) or by comparing the class of undernourished residents with that of the undernourished ($p=0.60$) or by comparing the class of obese residents to that of the non-obese ($p=0.55$). There was no significant difference for the nutritional status according to the year of the assessment, whether in overall analysis of the four nutritional status classes ($p=0.97$) or by comparing the class of undernourished residents to that of non-undernourished ($p=0.85$) or by comparing the class of obese residents to that of non-obese ($p=0.13$).

Discussion

This study is the first in France which gives exhaustive results on the nutritional status of residents in NH. It is not possible to claim that it is representative of all French nursing homes, but it nevertheless seems robust. Indeed, the average age of residents was close to that of all French NH (85.3 and 86.4 years respectively) (24) and the same was true for the sex ratio (respectively 70.6 and 73.6% of women) (24) and the prevalence of dementia (45.7 and 40.0% respectively) (24). Finally, the study covered all residents of the establishments explored, and this exhaustiveness was only found in one French study, but which related to a single nursing home (18).

In our study, the prevalence of undernutrition was probably underestimated, due to the fact that albuminemia was not taken into account. This prevalence (27.5%) corresponds to the estimate in France, which is 14.5 to 60.8% (17, 18) (Table 1). In a previous French study(17), the threshold

for BMI was 24, and undernutrition was samely overestimated. The difference with the prevalence of undernutrition in the 2012 French survey in the general population is major, since this figure was 2.6% for those aged 80 and over (10), but this survey focused on elderly people at home, a priori in better health than people in NH. Undernutrition is therefore an important problem in NH in France.

At an international level, the range of prevalence is even wider (1.5-71%) (Table 1), probably linked to very different populations and the use of equally different tools and thresholds for defining undernutrition. One recent study carried out in the USA have found a prevalence of less than 10% (9), the criterion was the existence of a BMI<18.5, with high prevalence of obesity as a counterpoint.

The higher prevalence of undernutrition in women appeared to be related to older age and the higher frequency in women with dementia and very low mobility. The increase in the risk of undernutrition in women that we have found is not always reported (2,13,25). Given the predominance of women in nursing homes, and the frequency of co-morbidities in them, we can think that additional studies could be interesting on this subject.

There are many explanations for the link between dementia and undernutrition. Indeed, there may exist in demented residents, anorexia, food refusal, difficulty in eating related to disorders of recognition of food or cutlery, an increase in the energy expenditure of physical activity in the walking dementia (12,26,27). Our study showed that demented residents had lower mobility than non-demented people, and yet were less often overweight, which suggests, for the population studied, that food was insufficient. Overall, the study seems to indicate that, for demented residents and as already pointed out, good availability of staff, careful nutritional monitoring and early management of undernutrition are necessary (26,28,29). Regarding the association between very

low mobility and the presence of undernutrition, our results are in agreement with other studies (28,29). Of course, the relationship between the two criteria can be understood in both directions: undernutrition can lead to sarcopenia, with a reduction in mobility, and low mobility can limit autonomy and the possibilities of eating. The higher prevalence of dementia may be a major reason, but other intercurrent conditions conventionally providing undernutrition (infections, pain, depression, neurological diseases, etc.) can lead to a decrease in mobility and therefore interfere.

The prevalence of obesity among the residents we studied was 22.9%. This result seems robust, because patients with BMI in an obesity zone, but who had an involuntary weight loss classifying them as undernourished were not counted as obese. To our knowledge, there is only one French study on obesity in NH, which is neither representative nor exhaustive (17), and which reports 27.4% of obese residents in a selected population of 346 residents. Recent international results, mainly from the USA, indicate a prevalence range of 15 to 30.7% (Table 1). In addition, according to the 2012 French survey of the general population, 16% of people aged 80 and over were obese (10). These results show that obesity is probably more frequent in nursing homes than in the general population of elderly people and therefore should not be underestimated. Indeed, it can increase the risk of morbi-mortality in the elderly (5) and, as in non-elderly adults, it can cause complications that lead to a reduction in quality of life and overuse of drugs (8). The increase in the prevalence of obesity in nursing homes observed in the USA (9,11) can obviously affect European countries, with important consequences in terms of costs, linked to the increase in needs in personnel and adapted materials (9,11). This should be the subject of reflection within health systems.

The absence of differences in the nutritional status of residents over the seven-year period considered suggests that the problems do not seem to vary over time in the region studied. For

obesity, in particular, the situation seems stable, in contrast to the data in the USA (9). It would however be interesting to repeat this study annually on a longer period.

The study has several limitations. In fact, 17.0% of residents could not be assessed either because of their refusal, or because of their very fragile condition. It was therefore not possible to compare residents excluded from included. However, the excluded were probably seriously handicapped, since the NH where the exclusions were the most numerous (n=45) was also the only one that received almost only dementias. There is therefore a good chance that exclusions, combined with the impossibility of obtaining an albuminemia have reduced the prevalence of undernutrition. Likewise, the prevalence of dementia and poor physical activity were likely to be reduced. It is more likely that finer criteria could have been used, in particular regarding patient mobility. An ADL score (30) would have given more precise elements. Regarding dementias, the seriousness of the attack has not been recorded. These details could not be obtained for reasons of availability of the evaluation staff, or else, if these elements were present in the residents' files, their time lag made them unusable. Finally, for reasons of team availability and access to files, the study was unable to integrate several factors of clinical condition as well as biological data from residents in NH. This is the main reason why we did not carry out a multivariate analysis of the criteria linked to undernutrition or obesity.

Conclusion

The nutritional screening carried out in 13 NH with 866 residents revealed undernutrition in 27.5% of cases and obesity in 22.9% cases, in the ranges of the usual values in NH. Undernutrition and obesity in NH are notable problems in France, compared to elderly people at home. The consequences in terms of staff workload and costs are probably significant. The women were older, more often malnourished, demented and with very limited mobility than the men. Undernutrition

and low mobility were significantly higher among those with dementia versus those without dementia. Very low mobility was higher among undernourished.

Ethical Approval and Consent to participate : The data were collected within the framework of the agreements signed between the Limousin Nutrition network (LINUT), health network of the Nouvelle Aquitaine Regional Health Agency and the Elderly Nursing Homes, which stipulate that patient data can be used for research purposes after approval by the patients or their legal representatives.

Consent for Publication: According to the agreement signed between the Limousin Nutrition network (LINUT), health network of the Nouvelle Aquitaine Regional Health Agency and the Elderly Nursing Homes, data collected with the approval of patients or their legal representatives can be used for publication.

Availability of supporting data: Under the agreements signed between Elderly Nursing homes and the Limousin Nutrition network (LINUT), health network of the Nouvelle Aquitaine Regional Health Agency, data are collected, saved and protected within the LINUT network.

Conflict of Interest (COI) Statement: Authors had no financial or personal relationships with companies or organizations sponsoring the research at the time it was carried out.

Funding: None

Authors' contributions:

Philippe Fayemendy, Jean-Claude Desport and Pierre Jesus designed the research; Philippe Fayemendy, Jean-Claude Desport, Aude Massoulard-Gainant, Carole Villemonteix and Pierre Jesus conducted the research; Jean-Claude Desport, Aude Masoulard and Carole Villemonteix

provided essential materials (databases); Jean-Claude Desport performed statistical analysis; Philippe Fayemendy, Jean-Claude Desport, Gustave Mabiama, Thibault Vernier, Pierre Jesus wrote the paper; Philippe Fayemendy, Gustave Mabiama, Jean-Claude Desport and Pierre Jesus have primary responsibility for the final content. All authors read and approved the final manuscript.

Acknowledgements: We thank the LINUT network, caregivers and residents of nursing homes of Limousin territory.

Authors' information

[1]Nutrition Unit and Specialized Centre for Obesity of the Limousin territory, University Hospital, avenue Martin Luther King, 87042, Limoges cedex, France

[2]Inserm U1094, Limoges university, IRD, Tropical Neuroepidemiology, Epidemiology and Neurology Tropical Institute, GEIST, rue du Dr Marcland, 87000 Limoges, France

[3]Health Regional Agency network Limousin Nutrition (LINUT), 16 rue du Cluzeau ; 87270 Isle, France

87042, Limoges cedex, France

[4]Microbiology, Immunology-Hematology and Morphologic Sciences Laboratory (LMIHSM), Doctoral Training Unit in Health Sciences (UFD-SCS), Doctoral School, Douala University, BP 2701, Douala, Cameroun

[5]Home hospitalization and Geriatric Service, University Hospital, avenue Martin Luther King,

References

1. Institut national de la statistique et des études économiques (Insee). Tableau de l'économie française, édition 2019, personnes âgées dépendantes, mars 2019. https://www.insee.fr/fr/statistiques/3676717?sommaire=3696937.
2. Challa S, Sharkey JR, Chen M, Phillips CD. Association of residents, facility, and geographic characteristics with chronic undernutrition in a nationally represented sample of older residents in US nursing homes. J Nutr Health Aging 2007;11(22):179-184..
3. Chan M, Lim YP, Ernest A, Tan TL. Nutritional assessment in an asian nursing home an dits association with mortality. J Nutr Health Aging 2010;14:23-28.
4. Raynaud-Simon A. Stratégie de dépistage et de prise en charge de la dénutrition: personne âgée. In: Traité de Nutrition Clinique, SFNEP ed., Paris, 2016:687-694.
5. Janssen I, Mark AE. Elevated body mass index and mortality risk in the elderly. Obes Rev 2007;8:41-59.
6. Villareal DT, Apovian CM, Kushner RF, Klein S. Obesity in older adults: technical review and position statement of the American Society for Nutrition and NAASO, the Obesity Society. Am J Clin Nutr 2005;82:923-934.
7. Bradway C, DuResta J, Fleshner I, Polomano RC. Obesity in nursing homes: a critical review. J Am Ger Soc 2008;56:1528-1535.
8. Felix HC. Safety of obese persons in nursing homes. Med Care 2018;56:1032-1034.
9. Zhang N, Field T, Mazor KM, Zhou Y, Lapane KL, Gurwitz JH. The Increasing Prevalence of Obesity in Residents of U.S. Nursing Homes: 2005-2015. J Gerontol A Biol Sci Med Sci. 2019 Nov 13;74(12):1929-1936. doi: 10.1093/gerona/gly265. PMID: 30689774.

10. Inserm, Kantar Health, Roche. ObEpi 2012. Enquête épidémiologique nationale sur le surpoids et l'obésité. 2012 https://www.roche.fr/content/dam/rochexx/roche-fr/roche_france/fr_FR/doc/obepi_2012.pdf.

11. Marihart CL, Brunt AR, Geraci AA. The high price of obesity in nursing homes. Care Manag J 2015;16:14-19.

12. Galesi LF, Leandro-Meri VA, Marques de Oliveira MR. Association between indicators of dementia and nutritional status in institutionalised older people. Int J Older Peop Nurs 2012;8:236-43.

13. Tamura BK, Bell CL, Masaki KH. Factors associated with weight loss, low BMI, and malnutrition among nursing home patients: a systematic review of literature. J Am Dir Assoc 2013;14:649-655.

14. Favaro-Moreira N, Krausch-Hofmann S, Matthys C, Vereeken C, Vanhauwaert E, Declercq A, Bekkering GE, Duyck J. Risk factors for malnutrition in older adults : a systematic review of the literature based on longitudinal data. Adv Nutr 2016;7:507-522.

15. Vandewoude MFJ, van Wijngaarden JP, De Maesschalck L, Luiking YC, Van Gossum A. The prevalence and health burden of malnutrition in Belgian older people in the community or residing in nursing homes: results of the NutriAction II study. Aging Clin Exp Research 2019;31:175-183.

16. Törmä J, winblad U, Cederholm T, Saletti A. does undernutrition still prevail among nursing home residents? Clin Nutr 2013:32:562-568.

17. Jesus P, Desport JC, Massoulard A, Villemonteix C, Baptiste A, Gindre-Poulvelarie L, Lorgueilleux S, Javerliat V, Fraysse JL, Preux PM. Nutritional assessment and follow-up of residents with and without dementia in nursing homes in the Limousin region of France: a health network initiative. J Nutr Health Aging 2012;16:504-508.

18. Desbordes F, Jouveau S, Broussaudier A, Noailletas V, Fayemendy P, Morin B, Misset B, Rebière F, Desport JC, Jésus P. État nutritionnel des résidents d'un établissement d'hébergement pour personnes âgées dépendantes (EHPAD) et liens entre la dénutrition, la consommation de compléments nutritionnels oraux et le service à l'assiette. Nutr Clin Metabol 2018;32:225-9.
19. Desport JC, Fraysse JL, Massoulard A, Gouraud M, Marchaisseau E, Philippon S, Villemonteix C, Ngoungou E-B, Dalmay F, Van Eunen S, Preux PM. LINUT: un réseau de nutrition pour les personnes âgées. Nutr Clin Metabol 2006;20:142-144.
20. Chumlea WC, Guo S, Wholihan K, Cockram D, Kuczmarski RJ, Johnson CL. Stature prediction equations for elderly non-hispanic white, non-hispanic black, and mexican american persons developed from NHANES III Data. J Am Diet Assoc 1998;98:137-142.
21. Haute Autorité de Santé. Stratégie de prise en charge en cas de dénutrition protéino-énergétique chez la personne âgée. Saint-Denis La Plaine: HAS; 2007. https://www.has-sante.fr/portail/upload/docs/application/pdf/denutrition_personne_agee_2007_-_recommandations.pdf
22. Agence nationale d'accréditation et d'évaluation en santé. Évaluation diagnostique de la dénutrition protéino-énergétique des adultes hospitalisés. Paris: ANAES; 2003. https://www.has-sante.fr/portail/upload/docs/application/pdf/de nutrition_recos_2006_09_25__14_20_46_375.pdf
23. Folstein MF, Folstein SE, McHugh PR. Mini-Mental State: A practical method for grading the cognitive state of patients for the clinician. J Psychiat Res 1975;12:189-198.
24. Ministère des solidarités et de la santé. Direction de la recherche, des études, de l'évaluation et des statistiques (DREES). 728000 résidents en établissements d'hébergement pour personnes âgées en 2015. Etudes et résultats 2017;1015:1-8.

25. Blaum CS, Fries BE, Fiatarone MA. Factors Associated With Low Body Mass Index and Weight Loss in Nursing Home Residents. J Gerontol A Biol Sci Med Sci 1995;50:M162-168.

26. Gillette-Guyonnet S, Nourashemi F, Andrieu S, de Glisezinski I, Ousset PJ, Rivière D, Albarède JL, Vellas B. Weight loss in Alzheimer disease. Am J Clin Nutr 2000;7(suppl):637S-642S.

27. Engelheart S, Brummer R. Assessment of nutritional status in the elderly: a proposed function-driven model. Food Nutr Res 2018;62:1366-1372.

28. Salva A, Coll-Planas L, Bruce S, De Groot L, Andrieu S, Abellan G, Vellas B et al. Nutritional Assessment of Residents in Long-Term Care Facilities (LTCFs): Recommendations of the Task Force on Nutrition and Ageing of the IAGG European Region and the IANA. J Nutr Health Aging 2009;13:475-483.

29. Bell CL, Masaki KH, Amella EJ. Factors associated with weight loss, low BMI, and malnutrition among nursing home patients: a systematic review of the litterature. J Am Med Dir Assoc 2013;14:649-655.

30. Katz S, Downs TD, Cash HR, Grotz RC. Progress in the development of the index of ADL. Gerontologist 1970;10:20-30.

.

Table 1: Undernutrition and obesity among elderly in nursing homes in certain high-income countries

First author / year	Country	n	Age (ys)	Women (%)	Undernutrition tools and thresholds	Undernutrition (%)	Obesity tools and thresholds	Obesity (%)
Challa / 2007** [2]	USA	128 514		74.4	BMI<18.5	12.1		
Chan / 2010 [3]	Singapore	154	77	53.2	BMI<18.5<br>MNA<17	52<br>39		
Törmä / 2013 [16]	Sweden	172	86.3	69.8	MNA-SF<br>BMI<22	30<br>40.7		
Vandewoude / 2019 [15]	Belgium	2480	86.3	78	MNA SF	13.5		
Zhang / 2019**** [9]	USA	1 743 443 (2005) 1 517 872 (2015)			BMI<18.5<br>BMI<18.5	8.5<br>7.2	BMI>=30<br>BMI>=30	22.4 (2005)<br>27.9 (2015)
Jésus / 2012 [17]	France	346	87.9	83.4	BMI<24 or MNA™<17	53.5	BMI >= 29	27.4
Desbordes / 2018* [18]	France	248	88	78.2	BMI<21 or Alb<35 g/L or weight loss>= 5% 1 mo or >=10% 6 mo	39.5		

MNA™: Mini Nutritional Assessment™; SF: Short Form; BMI: Body Mass Index; n: number; mo: month

*newly admitted residents, **representative data, ****long stay residents (>=100d/y).

Table 2: Nutritional Status of residents based on gender, dementia, and mobility.

Nutritional status	Gender			Dementia			Mobility		
	Women (%)	Men (%)	p	With (%)	Without (%)	p	Very low (%)	Poor or good (%)	p
			**<0.0001**			**<0.0001**			**<0.0001**
Undernutrition	30.7	20.0	**0.01**	34.4	19.2	**<0.0001**	49.5	16.7	**<0.0001**
Normality	32.2	34.9	>0.05	32.2	32.2	>0.05	22.7	32.2	**0.01**
Overweight	15.0	20.4	0.058	12.9	19.9	**0.01**	7.9	20.5	**<0.0001**
Obesity	22.1	24.7	>0.05	19.5	28.5	**0.004**	19.9	25.5	>0.05

Statistical tests used: ANOVA (or Kruskal-Wallis); Bold values indicate p<0.05

Click here to access/download;Figure;Figure 1 Nutritional status of all residents assessed.docx

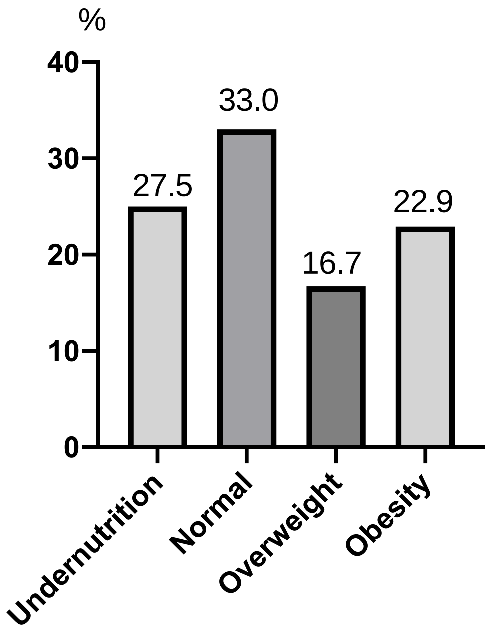

Figure 1: Nutritional status of all residents assessed
