# Supplementary material for: Nutritional status, dementia, and mobility among nursing home’s residents: First exhaustive cross-sectional study in Limousin territory (France)
Source: PLoS One. 2021 Apr 30;16(4):e0250595. doi: 10.1371/journal.pone.0250595 (PMC8087088; doi:10.1371/journal.pone.0250595)
Supplement: Attachment — Submitted filename: Response to Reviewers.docx [file pone.0250595.s002.docx]

Limoges, April 2^nd^ , 2021

From: Gustave Mabiama, corresponding Author

To: Reviewers

Sir,

My co-authors and I would like to thank you for reviewing the comments and suggestions made on our article. You will find in this letter the answers to the various points raised.

1. The study was approved by the Ethics Committee of the Regional Health Limousin Nutrition Network (LINUT) prior to data collection.
2. The Nursing Homes (NH) were all volunteers, they were contracted for clinical research with the LINUT network, and residents or their legal representatives gave their verbal informed consent for the assessments, noted on each residents’file by the LINUT dieticians.
3. Part of the work was presented by Pierre Jesus as e-poster (P419 - ASSESSMENT OF THE NUTRITIONAL STATUS OF 866 OLDER PEOPLE IN NURSING HOMES, AND LINKS WITH DEMENTIA AND MOBILITY) at the virtual congress on Clinical Nutrition and Metabolism organized by ESPEN from 19 to 21 September 2020. This e-poster published by Clinical Nutrition ESPEN 2020, 40, pp.590 does not value all aspects of the work done and results obtained, hence the idea of publishing the entire article in PLOS ONE.
4. The data are the property of LINUT network, a network of the Nouvelle Aquitaine Health Regional Agency. These data are linked with personal information of each resident followed by this network, and are not accessible, because they are considered as medical files. The restrictions were imposed by the network. Any request can be addressed to Dr Jean Louis Fraysse, 16 Rue du Cluzeau, 87170 Isle, [frayssejl@limut.fr](mailto:frayssejl@limut.fr).

Hoping you will agree with our responds.

Sincerely.

Gustave MABIAMA.

**Corresponding author**

Address correspondence: **Gustave MABIAMA**, Inserm U1094, Univ. Limoges, CHU Limoges, IRD, Tropical Neuroepidemiology, Institute of Epidemiology and Tropical Neurology, GEIST, Limoges, France 2 rue Dr Marcland, 87000 Limoges Cedex. E-mail address: [gustave.mabiama@unilim.fr](mailto:gustave.mabiama@unilim.fr); Phone: + 33 (0)6 05 89 88 35; Fax: + 33 5 55 05 63 54
